# Early in-hospital course of critically ill nontrauma patients in a resuscitation room of a German emergency department (OBSERvE2 study)

**DOI:** 10.1007/s00101-021-00962-3

**Published:** 2021-04-30

**Authors:** C. Grahl, T. Hartwig, L. Weidhase, S. Laudi, S. Petros, A. Gries, M. Bernhard

**Affiliations:** 1 Emergency Department, University Hospital of Leipzig, Leipzig, Germany; 2 Medical Intensive Care Unit, University Hospital of Leipzig, Leipzig, Germany; 3 Department of Anesthesiology and Intensive Care Medicine, University Hospital Leipzig, Leipzig, Germany; 4 Emergency Department, University Hospital of Düsseldorf, Heinrich-Heine-University, Moorenstraße 5, 40225 Düsseldorf, Germany

**Keywords:** Nontraumatic critically ill patients, Clinical pathway, Shock room, Emergency department, Mortality, Nicht-traumatische kritisch kranke Patienten, Klinischer Behandlungspfad, Schockraum, Notaufnahme, Mortalität

## Abstract

**Background:**

Management of critically ill nontrauma (CINT) patients in the resuscitation room of the emergency department (ED) is very challenging. Detailed data describing the patient characteristics and management of this population are lacking. This observational study describes the epidemiology, management and outcome in CINT ED patients in the resuscitation room.

**Methods:**

This prospective, single center observational study included all adult patients who were consecutively admitted to the ED resuscitation room during 2 periods of 1 year (September 2014–August 2015 vs. September 2017– August 2018). Patient characteristics, out-of-hospital/in-hospital treatment, admission-related conditions, time intervals for diagnostics and interventions and outcome were recorded using a self-developed questionnaire.

**Results:**

A total of 34,303 patients in the first and 35,039 patients in the second study period were admitted to the ED, of whom 532 and 457 patients, respectively, were admitted to the nontrauma resuscitation room due to acute life-threatening conditions. The patient characteristics did not differ significantly between the study periods (male: 58% vs. 59%, age: 68 ± 17 years vs. 65 ± 17 years). Time intervals for diagnostic and therapeutic interventions were similar. The CINT patients during the second study period were treated faster compared to the first study period (end of ED management: 53 ± 33 min vs. 41 ± 24 min, *p* < 0.0001). The 30-day all-cause mortality was comparable (34.0% vs. 36.3%).

**Conclusion:**

Observation of critically ill patient management in the ED resuscitation room showed reliable results between both study periods. Structured ED management guidelines for CINT patients may provide comparable results at one institution.

**Supplementary Information:**

The online version of this article (10.1007/s00101-021-00962-3) contains an additional table and the evaluation chart. The article and the supplementary material are available at www.springermedizin.de.

Please enter the article title in the search box; the supplementary material is available with the article under “Supplementary Information”.

## Abstract (de)

**Hintergrund:**

Das Management von nichttraumatologischen kritisch kranken Patienten im Schockraum einer Zentralen Notaufnahme (ZNA) ist sehr anspruchsvoll. Es fehlen detaillierte Daten, welche die Patientencharakteristika und das Management dieser Population beschreiben. Ziel der Beobachtungsstudie war es, die Epidemiologie, das Management und das Behandlungsergebnis bei nichttraumatologisch kritisch kranken Patienten im Schockraum zu beschreiben.

**Methoden:**

Diese monozentrische prospektive Beobachtungsstudie schloss alle erwachsenen Patienten ein, die während zweier 1‑Jahres-Perioden (September 2014 bis August 2015 vs. September 2017 bis August 2018) konsekutiv im Schockraum der ZNA aufgenommen wurden. Patientencharakteristika, prähospitale und innerklinische Maßnahmen, Aufnahmezustand, Zeitintervalle für Diagnostik und Interventionen sowie das Behandlungsergebnis wurden mittels eines selbst entwickelten Fragebogens erfasst.

**Ergebnisse:**

Insgesamt wurden 34.303 Patienten im ersten und 35.039 Patienten im zweiten Studienzeitraum in die ZNA aufgenommen, von denen 532 bzw. 457 Patienten aufgrund akut lebensbedrohlicher Zustände in den nichttraumatologischen Schockraum aufgenommen wurden. Die Patientencharakteristika unterschieden sich nicht signifikant zwischen den Studienzeiträumen (männlich: 58 % vs. 59 %, Alter: 68 ± 17 Jahre vs. 65 ± 17 Jahre). Die Zeitintervalle für diagnostische und therapeutische Interventionen waren ähnlich. Nichttraumatologische Patienten im zweiten Studienzeitraum wurden im Vergleich zum ersten Studienzeitraum schneller behandelt (Ende des Schockraummanagements: 53 ± 33 vs. 41 ± 24 min; *p* < 0,0001). Die 30-Tage-Gesamtmortalität war vergleichbar (34,0 vs. 36,3 %).

**Schlussfolgerung:**

Die Beobachtung des Managements kritisch kranker nichttraumatologischer Schockraumpatienten zeigt reliabel Ergebnisse im Vergleich beider Studienzeiträumen. Strukturierte Richtlinien zum Management kritisch kranker nichttraumatologischer Schockraumpatienten können vergleichbare Ergebnisse in einer Institution liefern.

## Treten Sie in den Austausch

Diese Arbeit wurde für *Der Anaesthesist* in Englisch eingereicht und angenommen. Die deutsche Zusammenfassung wurde daher etwas ausführlicher gestaltet. Wenn Sie über diese Zusammenfassung hinaus Fragen haben und mehr wissen wollen, nehmen Sie gern in Deutsch über die Korrespondenzadresse am Ende des Beitrags Kontakt auf. Die Autor*innen freuen sich auf den Austausch mit Ihnen!

## Background

Critically ill patients are globally mostly managed by emergency medical services (EMS) and thereafter are admitted to emergency departments (ED) [1, 2]. Early identification of the underlying problem and appropriate management after ED arrival improves outcome. Similar to the advanced trauma life support for the management of severely injured patients, critically ill patients with nontraumatic diseases in severe distress or shock are directly transferred to the resuscitation room as a special treatment area of an ED [1]. Nontraumatic critically ill patients treated in the resuscitation room showed a wide diversity of underlying diseases (e.g. sepsis, pneumonia, acute myocardial infarction, pulmonary embolism, cardiac arrest) [1]. In contrast to trauma, information on epidemiology, initial in-hospital approach and diagnostic procedures for patients suffering from nontraumatic conditions are scant [3, 4] and there is a lack of data on critically ill nontraumatic patients treated in resuscitation room of EDs in Germany [1].

After the results of the first investigation [1] during a 1-year study period in a German university ED, it was not clear whether these results were valid and reproducible in a given time interval. In order to standardize pending recommendations for nontraumatic critically ill patients, confirmation of the previous findings was considered essential.

The aim of this study was to evaluate nontraumatic critically ill resuscitation room patients in the ED during a second study period regarding the underlying epidemiology, out-of-hospital and in-hospital management, treatment intervals and outcome, as well as to compare these results with those from the first investigation at the same institution.

## Methods

From 1 August 2017 to 31 July 2018 we conducted a second prospective single-center observational study in a German university ED. The data from the first study period of 1 September 2014 to 31 August 2015 were obtained from the same ED and previously published [1]. Both study protocols were approved by the ethics committee of the University of Leipzig, Germany (264-14-25082014 and 198/17).

## Setting

More than 34,000 patients are managed annually in the ED of the University Hospital of Leipzig, with approximately half of them presenting with nontraumatic acute conditions or emergencies. The ED is part of a level I trauma center for the management of severely injured patients with a dedicated trauma team according to national recommendations [4]. The out-of-hospital management is provided by a two-tiered EMS system staffed with paramedics and EMS physicians. In our institution nontraumatic critically ill patients in the resuscitation room are managed by a team of two nurses, a resident and an attending physician with emergency and intensive care competency. Patients fulfilling the nontrauma resuscitation room activation criteria according to supplementary table S1 are admitted to the resuscitation room and the others are treated in a regular cabin of the ED. For patients with stable vital functions requiring urgent intervention, e.g. ST-elevation myocardial infarction or acute stroke, the ED is bypassed for percutaneous coronary intervention and computed tomography scan or thrombolytic treatment, respectively. There were essentially no staffing, space, or infrastructure changes in resuscitation room care procedures between the two study periods.

## Study definitions and data collection

All adult nontraumatic critically ill patients admitted to the ED resuscitation room were consecutively included. Pediatric and trauma patients were excluded. The team leader was responsible for the documentation of all aspects of the study using a self-developed evaluation chart (supplementary material chart 1). Documentation was based on the regular resuscitation room documentation and was completed immediately after the end of the resuscitation period. Missing data were incorporated after an interview between the team leader and the investigators and obtained from the patient records.

The ED resuscitation room evaluation chart included the patient characteristics (age, sex), risk scores (American Society of Anesthesiology, ASA score, National Advisory Committee of Aeronautics, NACA score) and the main reasons that led to ED resuscitation room admission according to the ABCDE approach.

The evaluation of the out-of-hospital EMS management included physician-to-physician communication by telephone before admission, airway management, noninvasive and invasive ventilation, catecholamines, intravenous or intraosseous access, 12-lead electrocardiogram (ECG), cardiopulmonary resuscitation, thrombolysis, induction of mild therapeutic hypothermia, chest tube insertion and automated external chest compression devices. The ED resuscitation room evaluation chart included the vital signs at resuscitation room arrival and resuscitation room discharge [systolic blood pressure (SBP, mm Hg), heart rate (HR, beat/min, bpm), oxygen saturation (pulse oxymeter, SpO2 (%), respiratory rate (x/min), shock index (SI, HF/SBP), capnopgraphy confirmed end-expiratory carbon dioxide (etCO2)]. The vital functions used as reference parameters were adapted from the existing literature: hypoxemia was defined as SpO2 less than 94% [5], bradycardia and tachycardia were defined as less than 60 bpm and more than 100 bpm, respectively [6], normotension was defined as between 100 mm Hg and 150 mm Hg and hypotension was defined as systolic blood pressure equal to or less than 90 mm Hg [7]. During the ED resuscitation room treatment period, definitive time points were recorded (e.g. admission time, end of handover, first systolic blood pressure measurement). Survival to discharge from resuscitation room and the signing out to the subsequent hospital ward/unit were recorded. Delay was defined as every additional minute of time spent in the ED resuscitation room after completion of the initial resuscitation period (e.g. boarding, imaging or subsequent nonresuscitation room-based intervention). The outcomes were all-cause mortality at day 30, and length of stay in the intensive care unit (ICU) and in the hospital.

## Statistical analysis

All items were collected using Microsoft Excel 2011 (Microsoft, Redmond, USA) and were analyzed by DataGraph 4.5.1 (Visual Data Tools). The descriptive statistics included numbers and percentages, mean ± standard deviation, median, and interquartile ranges. The χ^2^-test was applied for categorical data, and the Student’s t‑test for metric data. A *p* value <0.05 was considered to be statistically significant.

## Results

During the first and second 12-month study periods, 34,303 and 35,039 patients were admitted to the ED, respectively. In total 13,229 and 13,665 patients were excluded because of trauma as the leading diagnosis, including 592 (4.5%) and 625 (4.6%) patients, respectively, treated in the resuscitation room (as a substitute for then regular shock room) because of major trauma with trauma team activation. Out of the remaining 21,074 and 21,374 patients, 537 and 467 nontraumatic critically ill patients (2.5 and 2.2%) were admitted to the ED resuscitation room, respectively. In each study period 5 and 10 patients were excluded because of incomplete datasets. Data from 532 (99.1%) and 457 (97.9%) patients were available for final analysis.

## Patient characteristics

The patient characteristics are presented in Table 1. More men than women were admitted to the resuscitation room during both study periods. Men were significantly younger than women. The proportion of women aged 80 years and older was higher than that of men in both study periods.

**Table 1:** Patient characteristics

	First study period (*n* = 532) [1]		Second study period (*n* = 457)		*p*
Men, *n* (%) Women, *n* (%) Female: male	310 (58.3) 222 (41.7) 1: 1.4		273 (59.7) 184 (40.3) 1: 1.5		0.6556
*Age (years)*					1.0000
Mean ± SD	68 ± 17		65 ± 17		
Median (IQR)	71 (58–80)		66.5 (55–78)		
*Men, age (years)*					**0.0037**
Mean ± SD	67 ± 17		63 ± 16		
Median (IQR)	71 (57–78)		63 (55–76)		
*Women, age (years)*					0.0941
Mean ± SD	70 ± 17		67 ± 19		
Median (IQR)	74 (59–83)		67 (56–81)		
Women $\geq$80 years (%)	33.8	<0.0001	30.4	<0.0001	–
Men $\geq$80 years (%)	22.6		14.7		–
Weight (kg, mean ± SD) Median (IQR)	83 ± 31 80 (70–90)		81 ± 20 80 (70–90)		0.1272
Height (m, mean ± SD) Median (IQR)	1.7 ± 1.5 1.7 (1.7–1.8)		1.7 ± 1.5 1.7 (1.7–1.8)		1.0000
BMI (kg/m^2^, mean ± SD) Median (IQR)	28 ± 1 27 (24–31)		28 ± 7 26 (23–31)		1.0000
NACA score 4–6 (%)	92.0		85.0		**0.0005**
ASA classification 3–5 (%)	78.4		78.4		1.0000
*ABCDE problems, n (%)*					
A	20 (3.8)		17 (3.7)		0.9343
B	141 (26.5)		132 (28.8)		0.4199
C	189 (35.5)		160 (35.1)		0.8957
D	177 (33.3)		146 (31.9)		0.6399
E	5 (0.9)		2 (0.4)		0.3364
*Admitted by, n (%)*					
EMS	498 (93.6)		438 (93.7)		0.9488
In-hospital MET	25 (4.7)		6 (1.3)		**0.0022**
Interhospital transport	6 (1.1)		18 (3.9)		**0.0041**
ED triage	3 (0.6)		5 (1.1)		0.3881

*Mean* mean value, *SD* standard deviation, *IQR* interquartile range, *BMI* body mass index, *NACA* National Advisory Committee of Aeronautics, *ASA* American Society of Anaesthesiologist, *EMS* emergency medical service, *MET* medical emergency team, *ED* emergency department

The severity of illness assessed by the NACA score was slightly higher in the first study period compared to the second study according to the assessment of the EMS. A comparable proportion of patients in both study periods had pre-existing severe systemic disease conditions according to the ASA classification as assessed by the ED physician (Table 1).

According the ABCDE approach, indications for ED resuscitation room admission were comparable between both study periods, with circulatory distress and shock as the leading cause followed by unconsciousness, respiratory insufficiency, airway deterioration, and other issues (Table 1). In both study periods, most patients were brought in by EMS, followed by in-hospital medical emergency teams, interhospital critical care transport or by ED triage.

## Out-of-hospital and in-hospital emergency medical care

**Fig. 1:**
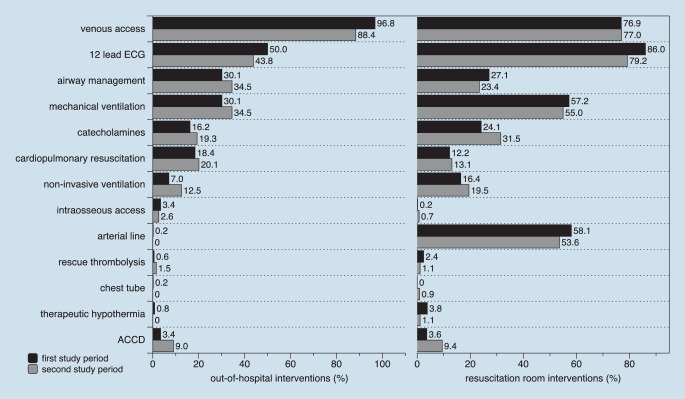
Out-of-hospital EMS and in-hospital resuscitation room interventions (*n* = 532 vs. *n* = 457). *ACCD* automated external chest compression devices; *EMS* emergency medical services. The data from the first study period were obtained from the same ED and previously published [1]

A comparison of the performed out-of-hospital EMS and ED resuscitation room-based emergency treatment is shown in Fig. 1. The proportion of performed interventions in the out-of-hospital setting between the first and the second study periods differed for intravenous access (96.8% vs. 88.4%, *p* < 0.0001), non-invasive ventilation (7.0% vs. 12.5%, *p* = 0.0033), and use of automated chest compression devices (ACCD, 3.4% vs. 9.0%, *p* = 0.0002). The proportion of performed interventions in the resuscitation room between the first and the second study period differed for 12-lead ECG (86.5% vs. 80.1%, *p* = 0.0068), catecholamine administration (24.1% vs. 31.5%, *p* = 0.0094), and use of ACCD (3.6% vs. 9.4%, *p* = 0.0002).

## Timeline of resuscitation room care

**Fig. 2:**
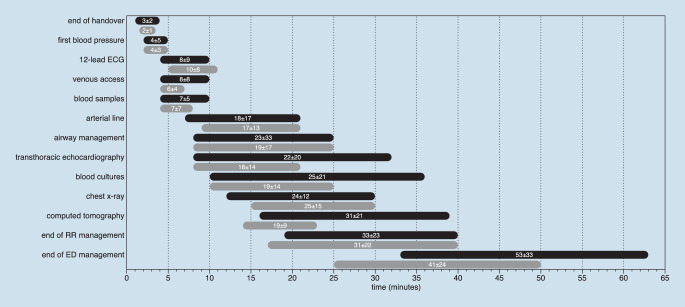
Time intervals between resuscitation room admission to medical interventions in minutes. Results were illustrated as interquartile ranges and mean ± standard deviation for the first (*black*) and second (*gray*) study period. *RR* resuscitation room, *ED* emergency department. The data from the first study period were obtained from the same ED and previously published [1]

Figure 2 and Table 2 show the time intervals between admission and different emergency interventions during the course of management for both study periods. Significant differences were found for end of handover, 12-lead ECG, venous access, and transthoracic echocardiography. Computed tomography was carried out more often in the first than in the second study period (42.7% vs. 36.5%, *p* = 0.0472). Of note, the time elapse from admission in the resuscitation room until the beginning of this diagnostic procedure was significantly shorter in the second than in the first study period (Table 2).

**Table 2:** Timeline of resuscitation room interventions

Time interval up to intervention (min)	First study period (*n* = 532) [1]	Second study period (*n* = 457)	*p*
End of handover, *n* (%)	532 (100)	457 (100)	*<0.0001*
Mean ± SD	3 ± 2	2 ± 1	
Median (IQR)	2 (1–4)	2 (2–3)	
First blood pressure, *n* (%)	519 (97)	408 (89)	1.0000
Mean ± SD	4 ± 5	4 ± 3	
Median (IQR)	3 (2–5)	3 (2–5)	
12-lead ECG, *n* (%)	459 (86)	362 (79)	*0.0009*
Mean ± SD	8 ± 9	10 ± 8	
Median (IQR)	5 (4–10)	9 (5–11)	
Venous access, *n* (%)	407 (77)	352 (77)	*<0.0001*
Mean ± SD	8 ± 8	6 ± 4	
Median (IQR)	6 (4–10)	5 (4–7)	
Blood samples, *n* (%)	496 (93)	383 (84)	1.0000
Mean ± SD	7 ± 5	7 ± 7	
Median (IQR)	6 (4–10)	6 (4–8)	
Blood cultures, *n* (%)	45 (8)	60 (13)	0.0823
Mean ± SD	25 ± 21	19 ± 14	
Median (IQR)	20 (10–36)	15 (10–25)	
Airway management, *n* (%)	141 (27)	107 (23)	0.2539
Mean ± SD	23 ± 33	19 ± 17	
Median (IQR)	15 (8–25)	13 (8–25)	
Arterial line, *n* (%)	307 (58)	245 (54)	0.4474
Mean ± SD	18 ± 17	17 ± 13	
Median (IQR)	15 (7–21)	15 (9–21)	
TTE, *n* (%) Mean ± SD Median (IQR)	149 (28) 22 ± 20 15 (8–32)	166 (36) 18 ± 14 15 (8–21)	*0.0390*
Chest x‑ray, *n* (%)	227 (43)	126 (28)	0.6374
Mean ± SD	24 ± 21	25 ± 15	
Median (IQR)	19 (12–30)	21 (15–30)	
Computed tomography, *n* (%)	227 (43)	167 (37)	*<0.0001*
Mean ± SD	31 ± 21	19 ± 9	
Median (IQR)	25 (16–39)	20 (14–23)	
End of RR management, *n* (%)	532 (100)	457 (100)	0.1645
Mean ± SD	33 ± 23	31 ± 22	
Median (IQR)	30 (19–40)	25 (17–40)	
End of ED treatment, *n* (%)	532 (100)	457 (100)	*<0.0001*
Mean ± SD	53 ± 33	41 ± 24	
Median (IQR)	45 (33–63)	36 (25–50)	

*ED* Emergency Department, *IQR* Interquartile range, *RR* Resuscitation Room, *TTE* transthoracic echocardiography, *MV* Mean Value, *SD* standard deviation

There was a delay until transfer to the next hospital ward after completion of all interventions in the resuscitation room in the first and the second study period in 100 (18.8%) and 63 (13.5%) patients, respectively, and this difference between the two study periods was significant (*p* = 0.02). In this subgroup, the mean ED stay was longer compared to patients without delay (82 ± 47 min vs. 47 ± 26 min, *p* < 0.001, and 76 ± 35 min vs. 68 ± 26 min, *p* < 0.001). In cases with delay, the additional mean waiting time interval was shorter in the first compared to the second study period (53 ± 34 min vs. 68 ± 57 min, *p* = 0.0368).

The lack of immediate ICU bed availability was the most common reason for delayed boarding (70.0%, *n* = 70 vs. 84.1%, *n* = 53, *p* = 0.0423), without a significant difference in time elapse between the two study periods (56 ± 50 min vs. 68 ± 57 min).

## Vital functions at hospital admission

On admission, mean SBP, SBP $\leq$90 mm Hg, mean HR, HR <60 or >100 bpm, mean SI, mean etCO2, mean respiratory rate, proportion of hypoxemic patients (SpO2 < 94%), and mean GCS did not significantly differ between the two study periods. Small but significant differences between the study cohorts were found for the proportion of hypotensive and hypertensive patients, mean SpO2, body temperature and the rate of cardiac arrest (Table 3).

**Table 3:** Vital functions at resuscitation room admission

	First study period (*n* = 532) [1]	Second study period (*n* = 457)	*p*
SBP, mm Hg (MV ± SD) Median (IQR)	135 ± 43 135 (102–162)	136 ± 40 137 (110–160)	0.7066
SBP < 100 and >150 mm Hg (%)	42.7	55.1	*0.0001*
SBP $\leq$90 mm Hg (%)	16.5	13.7	0.2217
HR, bpm (MV ± SD) Median (IQR)	96 ± 30 95 (80–115)	99 ± 32 98 (78–118)	0.1288
HR < 60 and >100 bpm (%)	44.4	46.1	0.5924
SI (MV ± SD, min) Median (IQR)	0.8 ± 0.5 0.7 (0.5–0.9)	0.8 ± 0.4 0.7 (0.6–1.0)	1.0000
SpO2, % (MV ± SD) Median (IQR)	92 ± 11 96 (88–100)	94 ± 10 97 (92–100)	*0.0030*
SpO2 < 94% (%)	39.5	36.5	0.3330
etCO2, mm Hg (MV ± SD) Median (IQR)	39 ± 16 35 (30–42)	40 ± 14 38 (34–47)	0.2997
RR, min^−1^ (MV ± SD) Median (IQR)	20 ± 10 16 (12–25)	20 ± 9 20 (12–25)	1.0000
GCS (MV ± SD) Median (IQR)	8 ± 5 8 (3–14)	8 ± 5 7 (3–14)	1.0000
Temp. tympanal, °C (MV ± SD) Median (IQR)	36.2 ± 1.4 36 (36.0–36.6)	36.5 ± 1.5 36.5 (35.0–37.2)	*0.0012*
Proportion of cardiac arrest without ROSC at hospital admission, *n* (%)	36 (36.4)	48 (52.7)	*0.0242*

*MV* Mean Value, *SD* standard deviation, *IQR* interquartile range, *SBP* systolic blood pressure, *HR* heart rate, *bpm* beats per minute, *SI* shock index, *RR* respiratory rate

## Diagnoses

**Fig. 3:**
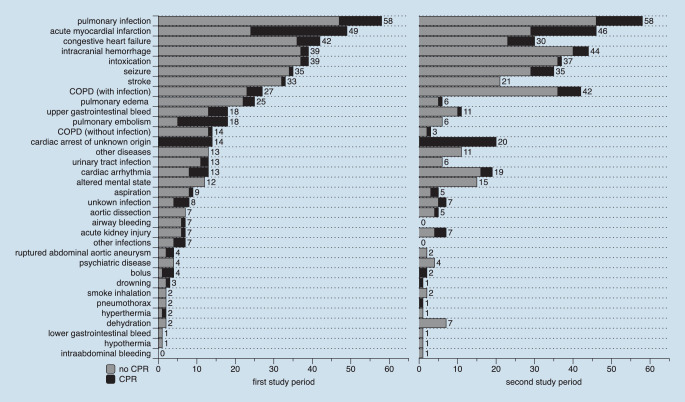
Diagnoses at hospital discharge or death for all patients (*n* = 532 vs. *n* = 457) including situations with (*black*) and without (*gray*) cardiac arrest. Numbers are per disease category. The data from the first study period were obtained from the same ED and previously published [1]

The major diagnoses verified at hospital discharge that led to ED admission are presented in Fig. 3. More than 50% of all life-threatening conditions in both study periods were related to deep airway infections (10.9% vs. 12.7%), acute myocardial infarction (9.2% vs. 10.1%), congestive heart failure (7.9% vs. 6.6%), intracranial bleeding (7.3% vs. 9.6%), poisoning (7.3% vs. 8.1%), seizure (6.6% vs. 7.7%) and cerebral ischemia (6.2% vs. 4.6%). These percentages did not significantly differ between the two periods.

During the first and second study period, 112 (21.1%) and 101 (22.1%) patients suffered from cardiac arrest during the out-of-hospital EMS or resuscitation room treatment, without significant difference. The origin of all cardiac arrests is shown in Fig. 3. Among the patients with out-of-hospital cardiac arrest (OHCA), 36/91 (36.4%) during the first study period, but 48/91 (52.7%) in the second period did not showed a return of spontaneous circulation at hospital admission (*p* = 0.0242). The figures for in-hospital cardiac arrest (IHCA) managed in the resuscitation room were similar for both study periods (11.6%, *n* = 13 vs. 9.9%, *n* = 10).

## Relocation site

After completion of resuscitation room management, the majority of patients were transferred to an intensive care (ICU) unit [84.5% (*n* = 450) in the first vs. 80.5% (*n* = 368) in the second period, *p* = 0.0361]. Prior to ICU admission, patients during the first study period were transferred less often to an interventional unit (e.g. angiography, cardiac catheterization laboratory, operating theatre) than during the second period [13.3% (*n* = 71) vs. 19.0% (*n* = 87), *p* = 0.0147)]. Furthermore, 9.9% (*n* = 53) vs. 11.4% (*n* = 52) of patients were admitted to general wards. Seven (1.3%) and four (0.9%) patients, respectively, were directly transferred for urgent cardiac surgery (e.g., aortic dissection). Less patients died during resuscitation room management during the first than the second study period [4.1% (*n* = 22) vs. 7.2% (*n* = 33), *p* = 0.0336].

## Outcomes

The 30-day mortality rate was comparable between the first and second study period (34.0% (*n* = 181) vs. 36.3% (*n* = 166)) (Table 4). The 30-day mortality in patients suffering from cardiac arrest at any time during the out-of-hospital or resuscitation room course was significantly higher than in patients without such an event (72.7 (*n* = 81/112) vs. 24.0% (*n* = 100/420), *p* < 0.001, for the first study period; and 79.2 (*n* = 80/101) vs. 24.2% (*n* = 86/356), *p* < 0.001, for the second period).

**Table 4:** Outcome of patients

	First study period (*n* = 532) [1]	Second study period (*n* = 457)	*p*
*30-day mortality*			
All patients, *n* (%)	181 (34.0)	166 (36.3)	0.4500
OHCA and IHCA, *n* (%)	81/112 (72.7)	80/101 (79.2)	0.2700
Without OHCA and IHCA, *n* (%)	100/420 (24.0)	86/356 (24.1)	0.9741
*ICU length of stay*			
OHCA and IHCA (days, MV ± SD)	6 ± 7	8 ± 11	0.1114
Without OHCA and IHCA (days, MV ± SD)	11 ± 10	13 ± 14	**0.0210**
*Hospital length of stay*			
OHCA and IHCA (days, MV ± SD)	7 ± 7	12 ± 14	**0.0038**
Without OHCA and IHCA (days, MV ± SD)	6 ± 8	8 ± 13	**0.0089**

*MV* Mean Value, *SD* standard deviation, *OHCA* out-of-hospital cardiac arrest, *IHCA* in-hospital cardiac arrest (during the resuscitation room management)

The hospital length of stay for patients without cardiac arrest was longer in the second than in the first study period (Table 4).

## Discussion

This prospective, single-center observational study (OBSERvE2 study) confirms for the first time the results of a previous investigation (OBSERvE study [1]) of nontraumatic critically ill patients treated in the resuscitation room of the same institution with respect to the underlying epidemiology, out-of-hospital and in-hospital management, treatment intervals and outcomes.

A comparable proportion of nontraumatic ED patients (2.5% vs. 2.2%) were treated in the resuscitation room in both study periods. The caseload of 532 and 457 nontraumatic critically ill patients in both study periods is comparable to the caseload of trauma team activation in the designated trauma center in the same institution. These findings demonstrated the need of the resuscitation room management of nontraumatic, critically ill patients, even if comparative numbers from other German EDs are not yet available. These findings support the same need for a 24/7 professional emergency medical care system and structured management guidelines, comparable to that for patients suffering from severe trauma [8].

Concerning the patient characteristics, patients in both study periods matched very well in terms of age and gender: men were more often treated than women, and one third of all women were 80 years and older. As known from previous trials of septic patients in the ED, a large proportion of the critically ill patients in our investigation were old, and men were more often treated than women [9–11]. Further findings, such as weight, height and body mass index (BMI) did not differ between the study periods. These findings demonstrate a high reliability and comparability between both study cohorts in the same institution in terms of epidemiology and patient characteristics.

The main reasons for resuscitation room admission were circulatory distress and hypotension (“C” problem), followed by unconsciousness (“D” problem) and respiratory insufficiency (“B” problem) in both groups of critically ill patients. In contrast to national recommendations for trauma patients [8], resuscitation room admission criteria for nontraumatic critically ill patients according to the ABCDE-approach are not implemented nationwide; however, our study group suggested resuscitation room criteria for nontraumatic critically ill patients in 2014 [12], and used them in the both study periods (supplementary material Table 1; [1]). From an evidence-based point of view, our previously described admission criteria seem to be suitable in order to gain balance between safety features and human and financial resources. Only 0.1% of patients in the first study period developed acute ABCDE deterioration during their ED stay [1].

The out-of-hospital and resuscitation room emergency interventions were comparable for both periods. One explanation for the small differences between the two study periods may be the systemwide introduction of Lund University Cardiopulmonary Assist System 2 (LUCAS‑2, Physiocontrol, Redmond, USA) as an ACCD in our EMS system. Even if the present investigation was not designed to study the introduction of an ACCD in the out-of-hospital setting, our findings underline that such a change in EMS corresponded to changes in the admitted patient cohort. Keeping this change in mind, we found a threefold higher rate of ACCD use in the out-of-hospital and in-hospital setting in the second study period. Furthermore, this change in the availability of ACCD led to a nearly twofold higher rate of patients in cardiac arrest state at hospital admission in the second in comparison to the first study period, resulting in a delayed implementation of 12-lead ECG and a shorter time interval up to transthoracic echocardiography. The more common use of ACCD and the associated changes in out-of-hospital resuscitation efforts may also be responsible for the higher proportion of patients who died at the end of resuscitation room management efforts in the second study period (4.1% vs. 7.2%, *p* = 0.0336). These findings were confirmed by previous investigation of our institution that showed that resuscitation time with ACCD is longer than with manual chest compression alone, resulting in a higher proportion of admitted patients [13]. The remaining emergency interventions in both investigated study periods were mainly comparable suggesting a highly standardized in-hospital care.

Comparing the time interval until emergency intervention, patients in both study periods were treated similarly. The timeline of emergency interventions in Fig. 2 suggested the parallelization of emergency procedures. Data from other German EDs are still lacking. Comparing the time elapse until computed tomography of 25 min (median) for trauma patients from the Trauma Registry of the German Trauma Society [14], our investigation showed a comparable resuscitation room management in trauma and nontrauma patients in terms of this diagnostic procedure.

The professionalization of emergency treatment in our institution over the years led to shorter time intervals and a quicker performance. In line with the literature, introduction of structured algorithm leads to better performance in resuscitation room management [3]. Moreover, a learning effect while performing resuscitation room management of nontraumatic critically ill ED patients over time may be observed in terms of shorter time intervals up to TTE, computed tomography, and end of ED treatment; however, the timeline of emergency interventions in both study groups represents only the performance in one institution. Therefore, multicenter trials should be conducted in order to confirm these findings.

The ED length of stay was prolonged in a small group of patients in both study periods, although the initial treatment was already completed. A significant finding in the first study period was that the main reason for delay was lack of immediate ICU bed capacity [1]. Discussing these findings in quality assurance rounds with all clinical institution of our hospital may have significantly reduced this proportion in the second study period by almost 50%.

A relevant proportion of patients in both study cohorts suffered from circulatory distress, unconsciousness and respiratory insufficiency. No relevant differences between both study cohorts were observed with respect to the diagnoses. Interestingly, there was similar proportion of pulmonary infections but a higher number of chronic obstructive pulmonary disease (COPD) with infection in the second study period. This may be explained by a stronger influenza B season in 2017/2018 compared to 2014/2015 [15].

The 30-day mortality rate in patients without cardiac arrest in the out-of-hospital and in-hospital setting with 24.0% and 24.1% were in line with previous investigations found for specific diseases, e.g. sepsis (24.4% [16]), septic shock (14.5–15.8% [9], 17.4–20.7% [10], 24.8–25.1% [11], 34.7% [16]), stroke (12.7–15.8% [17], 14.4% [18]), intracerebral hemorrhage (12.1–38.9% [19]) , and pneumonia (17.0% [20]).

In patients suffering from cardiac arrest, we found a 30-day survival rate of 27.8% and 20.8%, respectively. These findings are similar to the results of the European Registry of Cardiac Arrest (EuReCA TWO) study with 26.0% [21]. The trend towards a lower survival rate in the second study may be associated with the increasing use of out-of-hospital ACCD.

## Limitations

The major limitation of our investigation is that it is a single-center observational study; however, the reliability of the results from the first and the second study periods with over 450 enrolled patients in each study cohort provides valuable data on the resuscitation room management. Further multicenter studies should investigate the underlying epidemiology, out-of-hospital and in-hospital management, treatment intervals and outcomes of nontraumatic critically ill patients in the resuscitation room.

## Conclusion

Observation of critically ill patient management in the ED RR shows reliable results between both study periods. Structured ED management guidelines for nontrauma critically ill patients may provide comparable results at one institution.

## Supplementary Information

ESM 1_ Resuscitation room admission criteria

ESM 2_Evaluation chart

## Declarations

The study protocol was approved by the ethics committee of the Medical Faculty of the University of Leipzig, Germany (264-14-25082014 and 198/17). The requirement for consent to participate was waived by the ethics committee.
