# Supplementary material for: Early in-hospital course of critically ill nontrauma patients in a resuscitation room of a German emergency department (OBSERvE2 study)
Source: Anaesthesiologie. 2021 Apr 30;71(10):774–83. doi: 10.1007/s00101-021-00962-3 (PMC9525338; doi:10.1007/s00101-021-00962-3)
Supplement: Supplementary file 1 — ESM 1_ Resuscitation room admission criteria [file 101_2021_962_MOESM1_ESM.pdf]

Supplemental material **„Early In-hospital Course of Critically Ill Nontraumatic Patients in a Resuscitation Room of a German Emergency Department (OBSERvE2-study)" von Grahl C., Hartwig T., Weidhase L. et al. (2021) in *Der Anaesthesist.***

Article and additional material are available at www.springermedizin.de. Please enter the title of the article in the search field.

**Supplemental Table 1: Resuscitation room admission criteria*,[1]**
**Airway and breathing problems ("airway" and "breathing")**
- airway obstruction (e.g. tongue swelling)
- respiratory insufficiency with high respiratory rate (with respiratory weakness) or low oxygen saturation
- necessarity for invasive airway management
- invasive and non-invasive mechanical ventilation
**Circulation problems ("circulation")**
- cardiovascular insufficiency (e.g. hypotension, shock of any origin)
- state after or under cardiopulmonary resuscitation
- dysrhythmias
- bleeding
**Unconsciousness or neurological deficite ("disability")**
- ongoing unconsciousness of any origin
**Critical physical state ("environment")**
- intoxication with an ABCDE problem
- rhabdomyolysis
- hypothermia
*Additional other resuscitation room activation criteria may exist and activation depends on the attending physician in charge

[1] Bernhard M, Döll S, Hartwig T, et al. Resuscitation room management of critically ill nontraumatic-patients in a German emergency department (OBSERvE-Study). Eur J Emerg Med 2018; 25: e9-e17
