# Supplementary material for: Early in-hospital course of critically ill nontrauma patients in a resuscitation room of a German emergency department (OBSERvE2 study)
Source: Anaesthesiologie. 2021 Apr 30;71(10):774–83. doi: 10.1007/s00101-021-00962-3 (PMC9525338; doi:10.1007/s00101-021-00962-3)
Supplement: Supplementary file 2 — ESM 2_Evaluation chart [file 101_2021_962_MOESM2_ESM.pdf]

**Observation of critically ill patients in the Resuscitation Room of the Emergency Department (OBSERvE)-Study**

## Patient characteristics:

Age (year): ______ weight (kg): ___ height (cm): ___ O BMI: ___kg/m2
Gender: O male O female admission by: O EMS physician O EMS O self O MET
NACA: O I O II O III O IV O V O VI ASA: O I O II O III O IV O V O VI O VII

**Reason for resuscitation room admission:**

O cardiac arrest (diagnosis): ______________________________
O cardiac insufficiency/shock: o cardiac o septic o bleeding O other
(diagnosis): ______________________________
O respiratory failure, diagnosis: ______________________________
O gastrointestinal bleeding, diagnosis: ______________________________
O anaphylaxis, diagnosis: ______________________________
O sepsis, diagnosis: ______________________________
O neurological deficit: ______________________________
O unconsciousness, GCS __, diagnosis: ______________________________
O intoxikation, o CO o medication o drugs o alcohol, diagnosis: __________________
O trauma, type: ______________________________
O other reasons, diagonsis: ______________________________

## Out-of-hospital treatment:

Announcement prior admission by telephone: O yes O no O intubation of the trachea: O yes O no
O other airway device: __________ Indication given: O yes O no Rescue thrombolysis: O yes O no
O venous access: O yes O no IOA: O yes O no 12-lead-ECG: O yes O no O CPR: O yes O no
O chest tube: O yes O no MTH: O yes O no NIV: O yes O no catecholamines: O yes O no
O Problems in the OOH setting: ______________________________ ACCD: O yes O no

## Resuscitation room – Personnel / State ad admission:

disciplines: O ED O Anaesthesiology O Surgery O Internal Medicine O Neurology
O other: _________________ Number of ED Nurses: ___
O BP sys/dia __ / __ mmHg O HR __ / min O SpO2 __ % O Tymp Temp __°C O RR ___ /min
O at admission under ongoing CPR O yes O no O GCS: ____ O etCO2: ___ (if ventilated)

## Resuscitation Room – Time and Treatment:

O Admission: __ : __ h O end hand-over __: __ h O 1. BP O yes O no __ : __ h
O 12-lead-ECG O yes O no __ : __ h O blood sample O yes O no __ : __ h O BC O yes O no __ : __ h
O ETI oder SAD O yes O no __ : __ h O MV O yes O no __ : __ h O NIV O yes O no __ : __ h
O Capnograhy O yes O no __ : __ h O TTE O yes O no __ : __ h O chest x-ray O yes O no __ : __ h
O venous access O yes O no __ : __ h Number: __ Success O yes O no
O IOA O yes O no __ : __ h Number: __ Success O yes O no O BT: O yes O no __ : __ h
O arterial line O yes O no __ : __ h Success O yes O no O Rescue thrombolysis O yes O no __ : __ h
O CPR O yes O no __ : __ h ___ min O ACCD O yes O no __ : __ h
O Defibrillation O yes O no Number: __ O MTH O yes O no __ : __ h
O Intervention: ______________________________ O yes O no __ : __ h
O Intervention: ______________________________ O yes O no __ : __ h
O End initial treatment __ : __ h O other diagnostics (z.B. CT, MRI) O yes O no
other diagnostics:______________________________ start __ : __ h end __ : __ h
O 2$^{nd}$ hand-over __________ __ : __ h O transportation time ___ min

**Observation of critically ill patients in the Resuscitation Room of the Emergency Department (OBSERvE)-Study**

## Resuscitation Room – State ad end and relocation:

O BP sys/dia __ / __ mmHg O HR __ / min O SpO2 __ % O Tymp Temp __°C RR ___ /min
O CPR O yes O no time ____ min O ongoing CPR O yes O no O GCS: ____
O etCO2: ___ (if needed) O medical ICU (O ICU O IMC) O Stroke Unit O operative ICU
O OT O catheter lab O angio lab
O if resuscitation room end and further treatment in ED, admission to: ______________________
O survival to resuscitation room end: O yes O no Treatment time in resuscitation room: _____ min

## Resuscitation Room – Complication and treatment/admission delay:

complication 1: ___________________________
complication 2: ___________________________
complication 3: ___________________________
complication 4: ___________________________

delay 1: ______________________________________________ time: ____________ (min)

delay 2: ______________________________________________ time: ____________ (min)

delay 3: ______________________________________________ time: ____________ (min)

delay 4: ______________________________________________ time: ____________ (min)

## Outcome*:

O survival to ICU discharge O yes O no ICU LOS ____ days
O survival to hospital discharge O yes O no Hospital LOS ____ days

* added by study coordination

**Comments:**

________________________________________________________________________________

________________________________________________________________________________

________________________________________________________________________________

________________________________________________________________________________

Abbreviations:

ACCD Automated chest compression device, ASA American Society of Anesthesiologists Score, BP blood pressure, BC blood culture, BT blood transfusion, CO carbon monoxide, CPR cardiopulmonary resuscitation, CT computer tomography, ED emergency department, GCS Glasgow Coma Scale, HR heart rate, IOA intraosseous access, ICU intensive care unit, LOS length of stay, MET Medical emergency team, MRI magnetic resonance imaging, MTH mild therapeutic hypothermia, NACA National Advisory Committee of Aeronautics Score, NIV non-invasive ventilation, OOH out-of-hospital, OT operation theatre, RR respiratory rate, Temp tymp temperature tympanal

**OBSERvE**
